# Factor V Leiden 1691G > A mutation and the risk of recurrent pregnancy loss (RPL): systematic review and meta-analysis

**DOI:** 10.1186/s12959-020-00224-z

**Published:** 2020-06-24

**Authors:** Mohammad Masoud Eslami, Majid khalili, Mina Soufizomorrod, Saeid Abroun, Bahman Razi

**Affiliations:** 1 Department of Hematology, Faculty of Medical Sciences, Tarbiat Modares University, North Kargar Av, Tehran, 14117 Iran; 2 Department of Basic sciences, Maragheh University of medical sciences, Maragheh, Iran; 3 Rahat Breach and Sleep Research Center, Tabriz University of Medical Sciences, Tabriz, Iran

**Keywords:** Recurrent pregnancy loss, Factor V Leiden, 1691G > A mutation, Meta-analysis, Meta-regression

## Abstract

**Background:**

Although numerous replication case-control studies have attempted to determine the association between Factor V Leiden (FVL) 1691G > A mutation and susceptibility to Recurrent pregnancy loss (RPL), there have been confliction among the results of various ethnic groups. To address this limitation, here we implemented first meta-analysis to provide with consistent conclusion of the association between FVL 1691G > A mutation and RPL risk.

**Methods:**

After a systematic literature search, pooled odds ratio (OR) and their corresponding 95% confidence interval (CI) were used to evaluate the strength of the association. Additionally, meta-regression analyses were performed to find potential source of heterogeneity.

**Results:**

In this meta-analysis, 62 studies, containing 10,410 cases and 9406 controls, were included in quantitative analysis. Overall population analysis revealed a significant positive association in the dominant (OR = 2.15, 95% CI = 1.84–2.50, *P* < 0.001), over-dominant (OR = 1.88, 95% CI = 1.61–2.19, *P* < 0.001), allelic (OR = 2.05, 95% CI = 1.79–2.35, *P* < 0.001), and heterozygote (OR = 1.97, 95% CI = 1.68–2.30, *P* < 0.001) models. Moreover, a significant association of dominant (OR = 3.04, 95% CI = 2.04–4.54, *P* < 0.001), over-dominant (OR = 2.65, 95% CI = 1.74–4.05, *P* < 0.001), and heterozygote (OR = 2.67, 95% CI = 1.81–4.22, *P* < 0.001) models was found in the Iranian population. The subgroup analysis indicated strong significant association in Asian, European, Africa population, and case-control studies but not in South Americans and cohort studies.

**Conclusion:**

The FVL 1691G > A mutation and the risk of RPL confers a genetic contributing factor in increasing the risk of RPL, particularly in Iranians, except for South Americans.

## Introduction

Recurrent pregnancy loss (RPL) is a heterogeneous disorder which affects women of reproductive age. Recently, The American Society of Reproductive Medicine has defined RPL as two or more than two failed pregnancies before the 20th week of pregnancy [1–3]. Overall, 1–5% of women during reproductive ages could be affected [4]. From pathophysiological point of view, RLP might be influenced by various items, such as genetic factors (chromosomal aberrations, genetic polymorphisms), infectious diseases, structural abnormalities of the uterus, coagulative disorders (thrombophilia), endocrinological problems (thyroid disease and diabetes), and immunological disease (autoimmune disorder and inflammatory diseases) [5–7]. With considering these factors, still approximately 40 to 50% of cases remained idiopathic [8].

Although pregnancy as a physiological condition is associated with a hypercoagulable state, and the contact between placenta and maternal circulation is crucial for the establishment of a successful pregnancy, but any abnormality in this circulation, especially abnormal blood clotting in the small placental blood vessels, may results in RPL [9, 10]. During last decades, thrombophilia attracted a lot of attention as a risk factor for RLP. Thrombophilia is characterized as a hemostatic disorder which leads to an increased tendency of thromboembolic processes. Classically, thrombophilia could be classified into acquired and inherited forms [11, 12]. In this regards, antiphospholipid syndrome is an established acquired thrombophilia factor which increase the risk of RPL. Among inherited factors, mutation in Factor V Leiden (FVL) of the FV gene, G20210A of the FII (prothrombin) gene, and C677T of the methylenetetrahydrofolate reductase (MTHFR) gene are believed to play a key role in pathogenesis of RPL [13, 14].

FVL mutation shows an autosomal dominant pattern which occurs by substitution of guanine by adenine (CGA--- > CAA) at the nucleotide 1691 in the exon 10. As a result of this missense mutation, arginine (Arg) at amino acid 506 is substituted with glutamine (Gln), leading to generation of FVL resistant to the activated protein C (APC). APC is a natural anticoagulant which in normal situation cleaves activated factor V at amino acid 506 and makes it inactive [15–20].

Studies have shown that FVL mutation increases the risk of venous thrombosis 7 times in heterozygote and 80 times in homozygote carriers. In addition, it has been reported that this mutation increases the risk of pre-eclampsia in FVL carriers [21, 22]. The exact mechanism that FVL mutation influence the etiology of RPL is a controversial issue and has not yet been divulged thoroughly, but several studies suggested that production of micro thrombosis could sediment in delicate placental blood vessels and cause placental infarction and subsequent maternal and fetal complications [23, 24].

In spite of all findings, still the exact association between FVL mutation and the risk RPL is unclear and several investigators worldwide try to clarify this question. Therefore, here we conducted the first and the most comprehensive meta-analysis on the association between FVL 1691G > A mutation and risk of RPL by exerting 62 studies encompassing 10,410 cases and 9406 health control to achieve more reliable conclusion.

## Methods

Ethical approval is not necessary for this meta-analysis. The current meta-analysis was conducted according to the Preferred Reporting Items for Systematic reviews and Meta-Analyses (PRISMA) statement [25], including publication search, study selection, inclusion and exclusion criteria, data extraction, quality assessment, and statistical analysis.

### Publication search

A comprehensive systematic search in the ISI Web of Science, Scopus, and PubMed/Medline databases was conducted to retrieve all publications evaluating the associations between FVL 1691G > A mutation and susceptibility to RPL prior to May 2020. The following combinations of key words were used: (“Miscarriage” OR “abortion” OR “pregnancy loss” OR “habitual abortion” OR “fetal loss” OR “Recurrent Pregnancy Loss”) AND (“Factor V Leiden” OR “FV Leiden” OR “1691G > A” OR “rs6025”) AND (“polymorphism*” OR “variant” OR “mutation” OR “genotype” OR “allele” OR “single nucleotide polymorphism” OR “SNP”). In spite of detailed search, a manual cross-check of eligible studies and reviews was carried out to include other potential studies. Original data in English language and human population studies were collected.

### Study selection

Primary search strategy generates 1266 studies that were exported into Endnote X8 software. The duplicated studies were removed and title & abstract of remaining studies were reviewed by two investigators and irrelevant studies were excluded. Full-text verification was performed if we could not classify studies based on title & abstract. Any disagreements during study selection were discussed and resolved by consensus.

### Inclusion and exclusion criteria

Studies considered eligible if they met the following inclusion criteria: a) Studies concerning the association between FVL 1691G > A mutation and susceptibility to recurrent pregnancy loss as the main outcome; b) Studies that their case group have recurrent pregnancy loss (two or more times of abortion); c) Studies with case-control and cohort design; d) Studies reporting sufficient data of genotype or allele frequency that could confer feasibility of calculating the odds ratios (ORs) and 95% confidence intervals (CIs). On the other hand, duplicates, case reports, book chapters, reviews, letter to editor, studies with insufficient data, and abstracts were all excluded.

### Data extraction and quality assessment

According to a standardized extraction form, the following data were independently extracted by two investigators: the first author’s last name, journal and year of publication, country of origin, ethnicity, allele and genotype frequency in cases and controls, mean or range of age, genotyping method, and total sample size of cases and controls. The third investigator finalized the extracted data, and potential discrepancies were resolved by consensus. For quality assessment of the included publications, the Newcastle-Ottawa Scale (NOS) was applied [26]. In this respect, studies with 0–3, 4–6 or 7–9 scores were of, respectively, low, moderate, and high-quality.

### Statistical analysis

Deviation from Hardy–Weinberg equilibrium (HWE) for distribution of the genotype frequencies was analyzed by χ2-test in the control group. The strength of the association between FVL 1691G > A mutation and RPL risk was evaluated by the pooled OR and its corresponding 95% CI. Different comparison models for FVL 1691G > A mutation were as follow: dominant model (AA+GA vs. GG), over-dominant model (GA vs. GG + AA), allelic model (A vs. G), and heterozygote (GA vs. GG). It should be noted that due to the AA genotype frequency of zero in both cases and controls, the recessive and homozygote models were not calculable. Presence of heterogeneity between included studies was estimated by Cochran’s Q-statistic (*P* value< 0.10 was considered as statistically significant) [27]. Besides, to report quantitative heterogeneity I-squared (I^2^) tests was used. The fixed-effected model (FEM) was used if P_Q-statistic_ > 0.10 or I^2^ was< 50%; otherwise, the random-effected model (REM) was applied. In order to assessed the predefined sources of heterogeneity among included studies, subgroup analysis and meta-regression analysis based on year of population, the continent of the study population, and genotyping method were performed. Additionally, sensitivity analysis was conducted in presence of heterogeneity [28, 29]. Publication bias was estimated by Begg’s funnel plots and Egger’s regression test (*P* value< 0.05 was considered as statistically significant) [30, 31]. The funnel plot asymmetry was assessed with the Egger’s test. Practically, in case of no evidence of publication bias, studies with high precision (large study effects) will be located near the average line, and studies with low precision (small study effects) will be spread equally on both sides of the average line; any deviation from this shape can indicate publication bias. The data analyses were carried out using STATA (version 14.0; Stata Corporation, College Station, TX) and SPSS (version 23.0; SPSS, Inc. Chicago, IL) software.

## Results

### Study characteristics

**Fig. 1:**
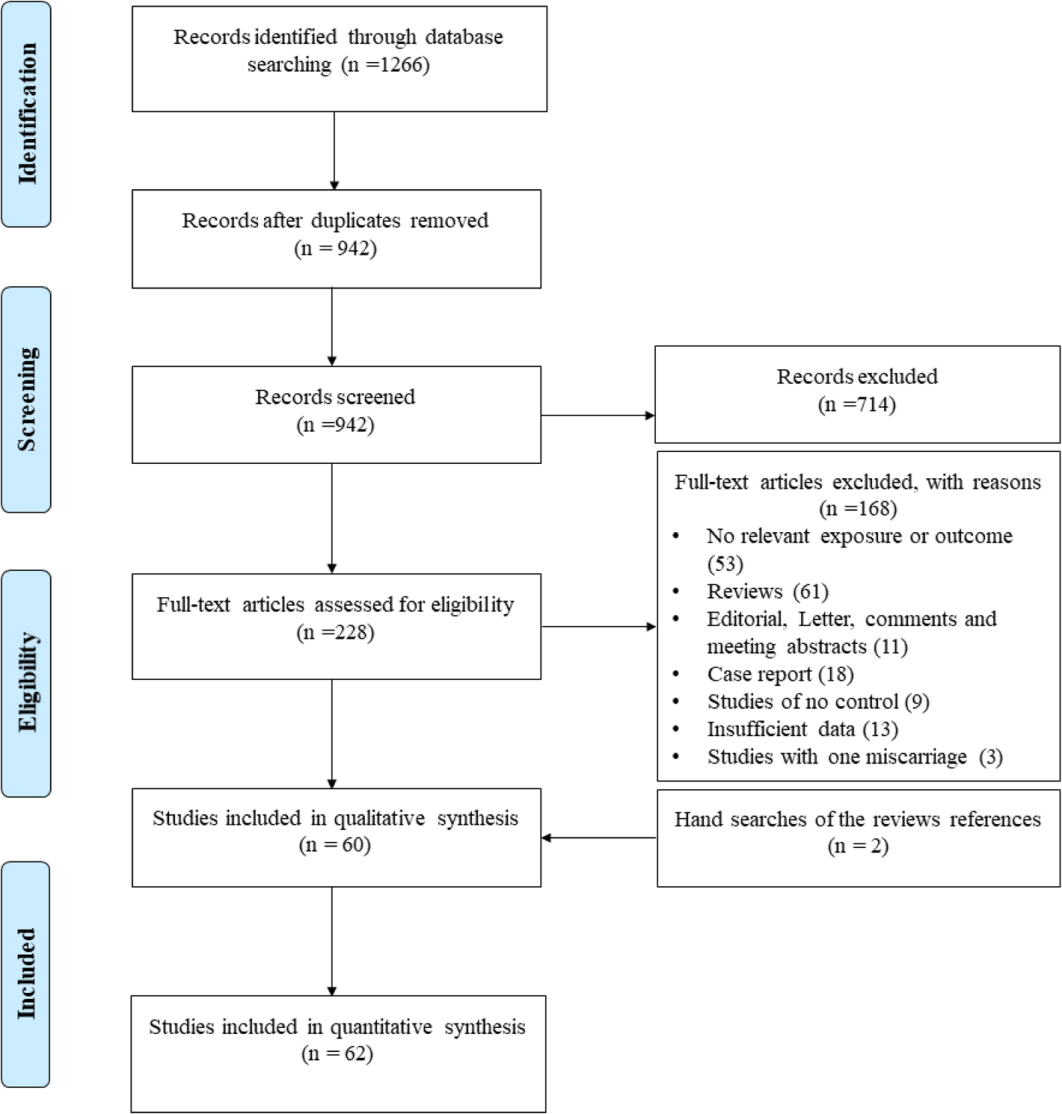
Flow diagram of study selection process

The four-phase search and screening process of the literatures based on the PRISMA statement is depicted in the Fig. 1. According to the aforementioned keywords, a total of 1266 studies were retrieved (PubMed: 254, Scopus: 512, and ISI Web of Science: 500). Subsequently, application of inclusion/exclusion criteria resulted in the exclusion of 1206 studies (324 duplicates studies, 714 and 168 studies excluded according to title & abstract and full-text examination, respectively). Eventually, 6**2** qualified studies were included in the quantitative analysis, of which two studies were detected by cross-check of eligible studies and reviews [32, 33]. All eligible studies were published between 1999 to 2019 and had an overall good methodological quality with NOS scores ranging from 5 to 8. The Restriction fragment length polymorphism (RFLP)-PCR was the most genotyping methods which used in the included studies. Except two studies which had cohort design, other **60** studies had case-control design. **Tables** **1** and **2** summarize the characteristics and allele/genotype frequency of the included studies.

**Table 1:** Characteristics of studies included in meta-analysis

Study author	Year	Country	Study design	Ethnicity	Total cases/controls	Age case/control (Mean)	Genotyping method	Quality score
Souza et al. [34]	1999	Brazil	case-control	South America	56/384	29.6 / 24.3	RLFP-PCR	7
Brenner et al. [35]	1999	Israel	case-control	Asia	76/106	31 ± 5 / 31 ± 6	RLFP-PCR	6
Wramsby et al. [36]	2000	Sweden	case-control	Europe	62/69	21–39 / 21–39	RLFP-PCR	7
Murphy et al. [37]	2000	Ireland	case-control	Europe	41/540	32 ± 0.74 / NR	RLFP-PCR	6
Pihusch et al. [33]	2000	Germany	case-control	Europe	102/128	35 / 32	RLFP-PCR	6
Younis et al.	2000	Israel	case-control	Asia	78/139	30.0 ± 4.4 / 30.7 ± 4.2	RLFP-PCR	6
Foka et al. [14]	2000	Greece	case-control	Europe	80/100	33 / 35	RLFP-PCR	6
Rai et al.	2001	London	cohort	Europe	1111/150	33.5 / 33	RLFP-PCR	8
Carp et al.	2002	Israel	case-control	Asia	108/82	31 / 36	RLFP-PCR	6
Finan et al. [38]	2002	Lebanon	case-control	Asia	110/67	32.3 ± 5.3 / 33.9 ± 7.3	RLFP-PCR	6
Hohlagschwandtner et al.	2003	Australia	case-control	Oceania	145/101	32 / 56	Multiplex PCR	7
Pauer et al. [39]	2003	German	case-control	Europe	30/122	31.3 / NR	RLFP-PCR	6
Mtiraoui et al	2004	Tunisia	case-control	Africa	146/99	29.0 ± 6.1 / 28.9 ± 5.3	RLFP-PCR	6
Aksoy et al.	2005	Turkey	case-control	Europe	41/50	32 ± 5.54 / 29 ± 4.66	PCR	5
Mahjoub et al. [40]	2005	Tunisia	case-control	Africa	200/200	28.68 ± 5.61 / 28.24 ± 5.51	RLFP-PCR	8
Ulukus et al.	2006	Turkey	case-control	Europe	10/53	29.1 ± 5.2 / 28.0 ± 4.8	PCR	5
Sotiriadis et al.	2006	Greece	case-control	Europe	99/102	32.2 / 32.2	RLFP-PCR	6
Mohammad et al. [21]	2007	Syrian	case-control	Asia	35/45	29.6 ± 6.3 / 28.8 ± 6.8	Q-PCR	5
Altintas et al. [41]	2007	Turkey	case-control	Europe	114/185	30.6 ± 4.4 / 30.5 ± 4.3	Q-PCR	7
Toth et al. [42]	2008	Germany	case-control	Europe	151/157	33.2 ± 4.6 / 45.2 ± 12.6	RLFP-PCR	7
Pasquier et al	2008	France	case-control	Europe	311/599	32.8 / 34.3	Q-PCR	8
Biswas et al. [43]	2008	India	case-control	Asia	85/31	27.9 ± 0.3 / 26 ± 0.5	RLFP-PCR	6
Lvanov et al.	2009	Bulgaria	case-control	Europe	153/100	29.7 / 31.0	RLFP-PCR	7
Mukhopadhyay et al. [44]	2009	India	case-control	Asia	84/80	24.9 ± 3.3 / 24.9 ± 3.3	RLFP-PCR	6
Ciacci et al. [45]	2009	Italy	case-control	Europe	39/72	36.24 ± 8.26 / 30.10 ± 8.60	Multiplex PCR	6
Mohamed et al. [46]	2010	Egypt	case-control	Africa	20/20	29.0 ± 4.80 / 31.4 ± 6.82	PCR	5
Hussein et al. [47]	2010	Palestine	case-control	Asia	145/205	31.9 / 32	ARMS-PCR	7
Serrano et al. [17]	2011	Portugal	case-control	Europe	100/100	32 ± 4.25 / 30.9 ± 5.19	PCR	7
Settin et al.	2011	Egypt	case-control	Africa	72/70	19 to 38 / 19 to 38	PCR	6
Dissanayake et al. [32]	2012	Sri Lanka	case-control	Asia	200/200	32.1 ± 5.6 / 32.4 ± 4.6	RLFP-PCR	8
Gazi et al.	2012	Turkey	case-control	Europe	57/47	30.12 ± 7.32 / 27.80 ± 6.36	PCR	6
Karata et al.	2012	Turkey	case-control	Europe	84/84	31.6 ± 3.7 / 32.2 ± 3.9	Q-PCR	6
Mierla et al. [48]	2012	Romania	case-control	Europe	283/100	33.76 / 32.8	RLFP-PCR	7
Ozdemir et al. [49]	2012	Turkey	case-control	Europe	543/106	27.8 ± 2.1 / 28.9 ± 2.2	Q-PCR	7
Torabi et al. [50]	2012	Iran	case-control	Asia	100/100	NR / NR	RLFP-PCR	6
Kaur et al.	2012	India	case-control	Asia	107/588	24.89 / 25.32	RLFP-PCR	7
Parveen et al.	2012	India	case-control	Asia	1000/500	28.4 ± 5.9 / 31.9 ± 7.3	ARMS-PCR	8
Ardestani et al.	2012	Iran	case-control	Asia	80/80	28.8 / 23.6	RLFP-PCR	6
Cardona et al. [51]	2012	Colombia	case-control	South America	93/206	34.1 ± 0.9 / 41.6 ± 0.7	RLFP-PCR	7
Kazerooni et al. [52]	2013	Iran	case-control	Asia	60/ 60	24.8 ± 3.9 / 24.6 ± 4.7	PCR	5
Baumann et al.	2013	Germany	cohort	Europe	641/157	32.95 ± 4.94 / 33.16 ± 6.24	RLFP-PCR	8
Parand et al. [53]	2013	Iran	case-control	Asia	90/44	29.21 ± 5.9 / 28.75 ± 5.2	RLFP-PCR	6
Zonouzi et al. [54]	2013	Iran	case-control	Asia	89/50	30.18 ± 4.95 / 31.54 ± 4.81	ARMS-PCR	6
Dutra et al.	2013	Brazil	case-control	South America	145/135	31.72 / 29.86	Q-PCR	6
Isaoglu et al.	2013	Turkey	case-control	Europe	60/40	29.14 ± 6.18 / 30.50 ± 6.77	NR	6
Pietropolli et al. [55]	2014	Italy	case-control	Europe	186/129	35.2 ± 5.1 / 40.4 ± 5.3	Rapid-cycle PCR	7
Lino et al.	2014	Brazil	case-control	South America	83/98	30.3 / 40.2	Q-PCR	6
Sharma et al. [56]	2015	India	case-control	Asia	78/78	28.6 ± 3.32 / 30.5 ± 2.57	RLFP-PCR	6
Farahmand et al.	2015	Iran	case-control	Asia	330/350	30.37 / 29.88	PCR	8
Kashif et al. [57]	2015	Pakistan	case-control	Asia	56/56	28.55 ± 4.69 / 28.61 ± 4.38	PCR	6
Gonçalves et al. [58]	2016	Brazil	case-control	South America	137/100	32.1 / 25.8	RLFP-PCR	7
Khaniani et al. [59]	2016	Iran	case-control	Asia	210/160	less than 40 / NR	RLFP-PCR	7
Eldeen et al.	2017	Arabia	case-control	Asia	96/96	37.7 ± 4.6 / 36.5 ± 5.8	PCR	6
Wolski et al. [60]	2017	Poland	case-control	Europe	359/400	30.99 ± 4.50 / 30.05 ± 3.81	RLFP-PCR	8
Elgari et al. [61]	2017	Arabia	case-control	Asia	60/80	38 ± 12 / 38 ± 12	Multiplex PCR	6
Mahmutbegović et al. [62]	2017	Bosnia	case-control	Europe	51/154	32.9 ± 5.1 / 31.7 ± 6.6	Q-PCR	6
Wingeyer et al.	2017	Argentina	case-control	South America	247/107	32 / NR	Q-PCR	7
Jusić et al.	2018	Bosnia	case-control	Europe	60/80	33.05 / 34.08	RLFP-PCR	6
Taghi Kardi et al.	2018	Iran	case-control	Asia	250/116	29.7 ± 3.4 / 30.4 ± 3.2	Multiplex PCR	7
Xu et al.	2018	China	case-control	Asia	426/444	29.26 ± 4.294 / 34.50 ± 4.895	Multiplex PCR	8
Bigdeli et al. [63]	2018	Iran	case-control	Asia	200/200	23.0 ± 3.8 / 25.1 ± 4.4	RLFP-PCR	8
Reddy et al. [64]	2019	India	case-control	Asia	50/28	26.8 / 27.6	RLFP-PCR	5
Yengel et al.	2019	turkey	case-control	Europe	145/105	30.5 ± 6.5 /30.5 ± 6.7	real-time PCR	6

**Table 2:** Distribution of genotype and allele among RPL patients and controls

Study author	RPL cases					Healthy control					P-HWE	MAF
	GG	GA	AA	G	A	GG	GA	AA	G	A		
Souza et al. [34]	52	4	0	108	4	378	6	0	762	6	0/87	0/007
Brenner et al. [35]	52	19	5	123	29	95	11	0	201	11	0/57	0/051
Wramsby et al. [36]	51	10	1	112	12	67	2	0	136	2	0/9	0/014
Murphy et al. [52]	39	2	0	80	2	527	13	0	1067	13	0/77	0/012
Pihusch et al. [33]	94	8	0	196	8	117	11	0	245	11	0/61	0/042
Younis et al.	63	12	3	138	18	131	8	0	270	8	0/72	0/028
Foka et al. [14]	65	15	0	145	15	96	4	0	196	4	0/83	0/02
Rai et al.	1037	72	2	2146	76	138	12	0	288	12	0/6	0/04
Carp et al.	104	4	0	212	4	77	5	0	159	5	0/77	0/03
Finan et al. [38]	65	38	7	168	52	56	11	0	123	11	0/46	0/082
Hohlagschwandtner et al.	130	15	0	275	15	97	4	0	198	4	0/83	0/019
Pauer et al. [39]	28	2	0	58	2	113	9	0	235	9	0/67	0/036
Mtiraoui et al.	116	24	6	256	36	93	6	0	192	6	0/75	0/03
Aksoy et al.	31	9	1	71	11	45	5	0	95	5	0/7	0/05
Mahjoub et al. [40]	152	40	8	344	56	189	11	0	389	11	0/68	0/027
Ulukus et al.	7	3	0	17	3	49	3	1	101	5	≤0.001	0/047
Sotiriadis et al.	94	5	0	193	5	99	3	0	201	3	0/88	0/014
Mohammad et al. [21]	25	10	0	60	10	41	4	0	86	4	0/75	0/044
Altintas et al. [41]	105	9	0	219	9	172	13	0	357	13	0/62	0/035
Toth et al. [42]	138	13	0	289	13	145	12	0	302	12	0/61	0/038
Pasquier et al.	296	15	0	607	15	574	25	0	1173	25	0/6	0/02
Biswas et al. [43]	83	2	0	168	2	31	0	0	62	0	≤0.001	0
Lvanov et al.	133	19	1	285	21	93	7	0	193	7	0/71	0/035
Mukhopadhyay et al. [44]	80	4	0	164	4	80	0	0	160	0	≤0.001	0
Ciacci et al. [45]	38	1	0	77	1	70	2	0	142	2	0/9	0/013
Mohamed et al. [46]	6	12	2	24	16	19	1	0	39	1	0/9	0/025
Hussein et al. [47]	104	36	5	244	46	181	24	0	386	24	0/37	0/058
Serrano et al. [17]	95	5	0	195	5	95	5	0	195	5	0/79	0/025
Settin et al.	54	17	1	125	19	69	1	0	139	1	0/95	0/007
Dissanayake et al. [32]	196	4	0	396	4	195	5	0	395	5	0/85	0/012
Gazi et al.	50	6	1	106	8	43	4	0	90	4	0/76	0/042
Karata et al.	66	16	2	148	20	66	18	0	150	18	0/27	0/107
Mierla et al. [48]	260	21	2	541	25	95	5	0	195	5	0/79	0/025
Ozdemir et al. [49]	433	109	1	975	111	104	2	0	210	2	0/92	0/009
Torabi et al. [50]	87	12	1	186	14	96	4	0	196	4	0/83	0/02
Kaur et al.	102	4	1	208	6	573	15	0	1161	15	0/75	0/012
Parveen et al.	950	50	0	1950	50	488	12	0	988	12	0/78	0/012
Ardestani et al.	78	2	0	158	2	79	1	0	159	1	0/95	0/006
Cardona et al. [51]	92	1	0	185	1	205	1	0	411	1	0/97	0/002
Kazerooni et al. [52]	43	12	5	98	22	54	4	2	112	8	0.48	0.734
Baumann et al.	592	49	0	1233	49	145	12	0	302	12	0/61	0/038
Parand et al. [53]	72	15	3	159	21	38	6	0	82	6	0/62	0/068
Zonouzi et al. [54]	87	2	0	176	2	50	0	0	100	0	≤0.001	0
Dutra et al.	142	3	0	287	3	131	4	0	266	4	0/86	0/014
Isaoglu et al.	47	13	0	107	13	39	1	0	79	1	0/93	0/012
Pietropolli et al. [55]	168	18	0	354	18	125	4	0	254	4	0/85	0/015
Lino et al.	79	4	0	162	4	96	2	0	194	2	0/91	0/01
Sharma et al. [56]	36	40	2	112	44	77	1	0	155	1	0/95	0/006
Farahmand et al.	302	28	0	632	28	340	10	0	690	10	0/78	0/014
Kashif et al. [57]	53	3	0	109	3	56	0	0	112	0	≤0.001	0
Gonçalves et al. [58]	133	4	0	270	4	98	2	0	198	2	0/91	0/01
Khaniani et al. [59]	202	8	0	412	8	158	2	0	318	2	0/93	0/006
Eldeen et al.	0	72	24	72	120	0	94	2	94	98	≤0.001	0/51
Wolski et al. [60]	333	26	0	692	26	378	21	1	777	23	0/23	0/028
Elgari et al. [61]	56	4	0	116	4	74	6	0	154	6	0/72	0/037
Mahmutbegović et al. [62]	44	7	0	95	7	142	12	0	296	12	0/61	0/038
Wingeyer et al.	239	8	0	486	8	105	2	0	212	2	0/92	0/009
Jusić et al.	51	9	0	111	9	77	3	0	157	3	0/86	0/018
Taghi Kardi et al.	236	12	2	484	16	109	5	2	223	9	≤0.001	0/038
Xu et al.	426	0	0	852	0	443	1	0	887	1	0/98	0/001
Bigdeli et al. [63]	150	30	20	330	70	192	8	0	392	8	0/77	0/02
Yengel et al.	130	1	14	261	29	102	0	3	204	6	0/65	0/394

*P-HWE p*-value for Hardy–Weinberg equilibrium; *MAF* Minor allele frequency of control group

### Meta-analysis of FVL 1691G > A mutation and the risk of RPL

**Fig. 2:**
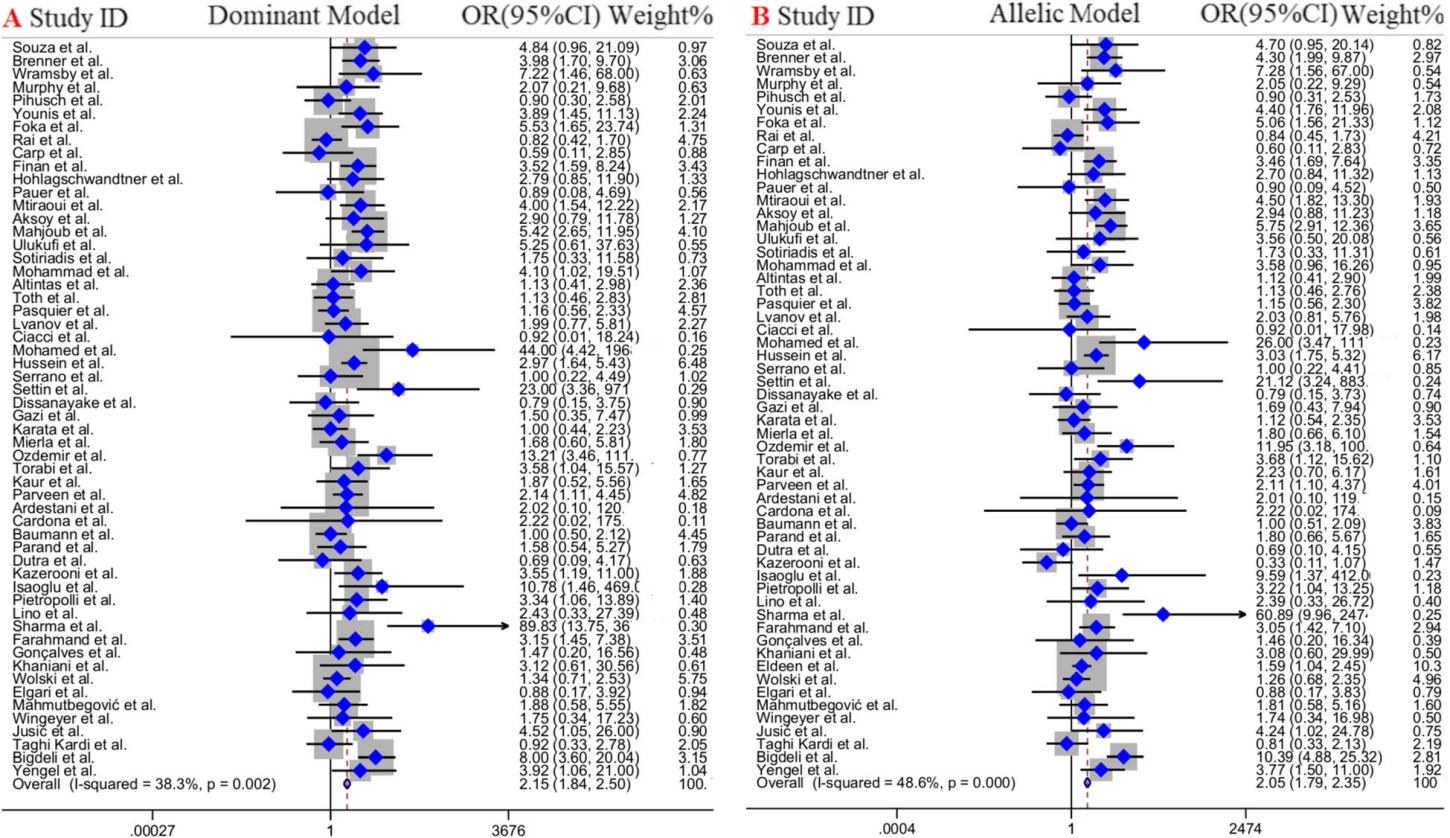
Pooled odds OR and 95% confidence interval of individual studies and pooled data for the association between FVL 1691G > A mutation and the risk of RPL in overall populations for **a**; Dominant Model, **b**; Allelic Model

Overall, 6**2** studies with 10,**410** cases and 9**406** controls included in quantitative analysis of the association between FVL 1691G > A mutation and the risk of RPL. Of those, 25 studies were in Asian countries [21, 22, 32, 35, 38, 43, 44, 47, 50, 52–54, 56, 57, 59, 61, 63–71], 2**6** studies were conducted in European countries [17, 33, 36, 37, 39, 41, 42, 45, 48, 49, 55, 60, 62, 72–82], 6 studies in South American countries [34, 51, 58, 83–85], 4 studies in African countries [40, 46, 86, 87] and one study in Oceania. The analysis of overall population revealed a significant positive association between FVL 1691G > A mutation and the risk of RPL across all possible genotype models, including dominant model (OR = 2.1**5,** 95% CI = 1.8**4**–2.**50,***P* < 0.001, FEM), over-dominant model (OR = 1.88, 95% CI = 1.61–2.19, *P* < 0.001, FEM), allelic model (OR = 2.05, 95% CI = 1.**79**–2.**35**, *P* < 0.001, REM), and heterozygote model (OR = 1.97, 95% CI = 1.68–2.30, *P* < 0.001, FEM) (Table 3 and Fig. 2).

**Table 3:** Main results of pooled ORs in meta-analysis of FVL 1691G > A mutation

**Subgroup**		**Sample size**	**Test of association**		**Test of heterogeneity**		**Test of publication bias (Begg’s test)**		**Test of publication bias (Egger’s test)**	
	**Genetic model**	**Case/Control**	**OR**	**95% CI (*P*-value)**	**OR**	**P**	**Z**	**P**	**T**	P
**Overall**	Dominant	10,410 / 9406	**2.15**	**1.84–2.50 (< 0.001)**	38.3	0.002	1.49	0.13	1.64	0.11
	Over-Dominant	10,410 / 9406	**1.88**	**1.61–2.19 (< 0.001)**	35.8	0.005	1.33	0.17	1.45	0.14
	Allelic model	10,410 / 9406	**2.05**	**1.79–2.35 (< 0.001)**	48.6	≤0.001	1.45	0.16	1.59	0.13
	GA vs. GG	10,410 / 9406	**1.97**	**1.68–2.30 (< 0.001)**	28.3	0.03	1.51	0.11	2.01	0.04
**Iranian population**	Dominant	1409 / 1160	**3.04**	**2.04–4.54 (< 0.001)**	37.3	0.13	−0.45	0.65	−0.53	0.61
	Over-Dominant	1409 / 1160	**2.65**	**1.74–4.05 (< 0.001)**	0	0.66	−1.05	0.29	−0.64	0.55
	Allelic model	1409 / 1160	2.09	0.88–4.94 (< 0.09)	76.8	0.008	−0.45	0.65	−0.33	0.75
	GA vs. GG	1409 / 1160	**2.67**	**1.81–4.22 (< 0.001)**	0	0.59	−1.05	0.29	−0.67	0.53
**Subgroup (continent)**										
**Asia**	Dominant	4153 / 3957	**2.80**	**2.20–3.56(< 0.001)**	35.4	0.06	−0.80	0.42	− 0.44	0.64
	Over-Dominant	4153 / 3957	**2.22**	**1.73–2.85 (< 0.001)**	47.2	0.01	−1.43	0.15	−0.98	0.34
	Allelic model	4153 / 3957	**2.35**	**1.92–2.87 (< 0.001)**	62.6	0.003	− 0.45	0.64	0.39	0.7
	GA vs. GG	4153 / 3957	**2.51**	**1.95–3.21 (< 0.001)**	11.9	0.31	−1.10	0.27	−0.35	0.73
**Europe**	Dominant	4913 / 3929	**1.49**	**1.20–1.84 (0.001)**	14.7	0.25	3.06	0.002	3.79	0.001
	Over-Dominant	4913 / 3929	**1.43**	**1.15–1.79 (0.002)**	16	0.23	2.99	0.003	3.65	0.001
	Allelic model	4913 / 3929	**1.48**	**1.19–1.81 (0.001)**	9.9	0.32	1.37	0.16	1.58	0.13
	GA vs. GG	4913 / 3929	**1.44**	**1.15–1.80 (0.001)**	16	0.23	2.96	0.003	3.65	0.001
**South America**	Dominant	761 / 1030	2.04	0.88–4.74 (0.09)	0	0.76	0.19	0.85	−0.53	0.62
	Over-Dominant	761 / 1030	2.04	0.88–4.74 (0.09)	0	0.76	0.19	0.85	−0.53	0.62
	Allelic model	761 / 1030	2	0.87–4.60 (0.1)	0	0.76	−0.19	0.85	−0.47	0.66
	GA vs. GG	761 / 1030	2.04	0.88–4.74 (0.09)	0	0.76	0.19	0.85	−0.53	0.62
**Africa**	Dominant	438 / 389	**5.65**	**3.15–10.14 (< 0.001)**	3.9	0.37	1.36	0.17	2.55	0.12
	Over-Dominant	438 / 389	**4.44**	**2.45–8.03 (< 0.001)**	3.2	0.37	1.36	0.17	2.41	0.13
	Allelic model	438 / 389	**5.93**	**3.38–10.40 (< 0.001)**	0	0.55	1.36	0.17	2.59	0.12
	GA vs. GG	438 / 389	**4.70**	**2.59–8.53 (< 0.001)**	12.3	0.33	1.36	0.17	2.47	0.13
**Subgroup (Study design)**										
**Case-Control**	Dominant	8658 / 9099	**2.33**	**1.99–2.74 (< 0.001)**	31.5	0.01	0.28	0.78	0.79	0.43
	Over-Dominant	8658 / 9099	**2.05**	**1.74–2.41 (< 0.001)**	29.3	0.02	1.43	0.15	1.28	0.21
	Allelic model	8658 / 9099	**2.18**	**1.8–2.52 (< 0.001)**	44.9	0.003	0.71	0.47	0.87	0.39
	GA vs. GG	8658 / 9099	**2.16**	**1.83–2.55 (< 0.001)**	18.2	0.13	1.70	0.09	1.78	0.08
**Cohort**	Dominant	1752 / 307	**0.90**	**0.55–1.49 (0.68)**	0	0.69	−1.23	0.47	−1.88	0.11
	Over-Dominant	1752 / 307	**0.88**	**0.54–1.46 (0.63)**	0	0.64	−1.23	0.47	−0.95	0.38
	Allelic model	1752 / 307	**0.91**	**0.56–1.49 (0.71)**	0	0.72	−1.23	0.47	− 1.27	0.25
	GA vs. GG	1752 / 307	**0.88**	**0.54–1.46 (0.63)**	0	0.64	−1.23	0.47	−1.68	0.14

### Meta-analysis of FVL 1691G > A mutation and the risk of RPL in Iranian population

Among the included studies, studies performed in Iran with 9 publications (1409 cases and 1160 controls) were in the first rank with respect to sample size and the number of studies, therefore we performed separate analysis. Our results found a significant association between FVL 1691G > A mutation and increased risk of RPL in this population under dominant model (OR = 3.04, 95% CI = 2.04–4.54, *P* < 0.001, FEM), over-dominant model (OR = 2.65, 95% CI = 1.74–4.05, *P* < 0.001, FEM), and heterozygote model (OR = 2.67, 95% CI = 1.81–4.22, *P* < 0.001, FEM) but not allelic model (OR = 2.09, 95% CI = 0.88–4.94, *P =* 0.09, REM) (Table 3).

### Subgroup analysis by continent

**Fig. 3:**
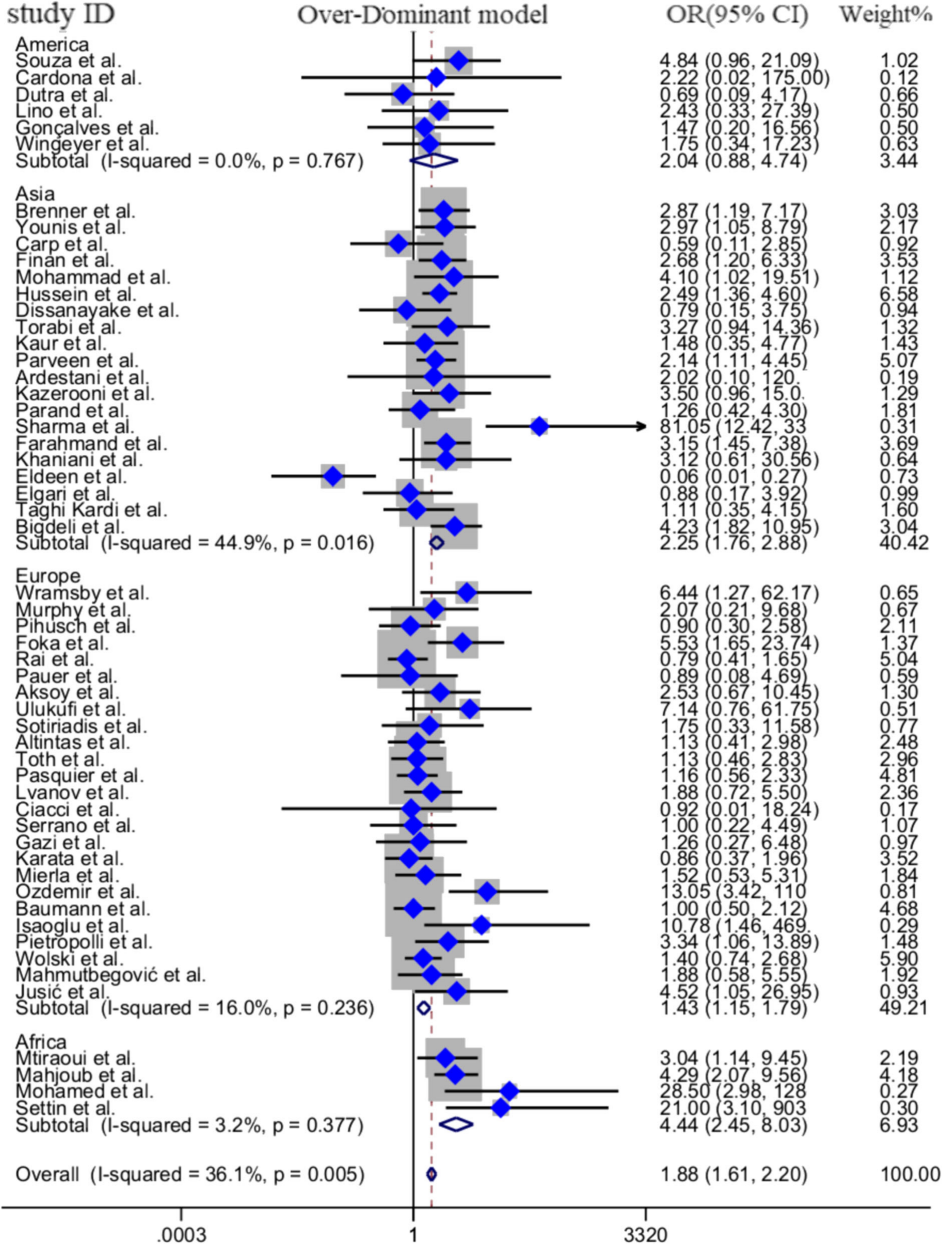
Pooled OR and 95% CI of individual studies and pooled data for the association between FVL 1691G > A mutation and the risk of RPL in different continents based on subgroup analysis for Over-Dominant model

The included studies were performed in Asia (25 studies), Europe (2**6** studies), South America (6 studies), Africa (4 studies) and Oceania (1 article). Since there was only one study for Oceania, we exclude it from the subgroup analysis. The final results revealed strong significant association between FVL 1691G > A mutation and the risk of RPL in Asian, European, and Africa population, but not in South Americans (Fig. 3). The results of pooled ORs, heterogeneity tests, and publication bias tests in different analysis models are shown in the Table 3.

### Subgroup analysis by study design

The stratification of studies based on study design caused to the inclusion of two studies with 1752 cases and 307 controls in cohort group, and 60 studies with 8658 cases and 9099 controls in case-control group. The findings demonstrated a statistical significant association between FVL 1691G > A mutation and the risk of RPL in case-control studies across dominant model (OR = 2.33, 95% CI = 1.99–2.74, *P* < 0.001, FEM), over-dominant model (OR = 2.05, 95% CI = 1.74–2.41, *P* < 0.001, FEM), allelic model (OR = 2.18, 95% CI = 1.8–2.52, *P* < 0.001, FEM), and heterozygote model (OR = 2.16, 95% CI = 1.83–2.55, *P* < 0.001, FEM). However, no significant association was observed in cohort studies (Table 3).

### Heterogeneity and publication bias

**Fig. 4:**
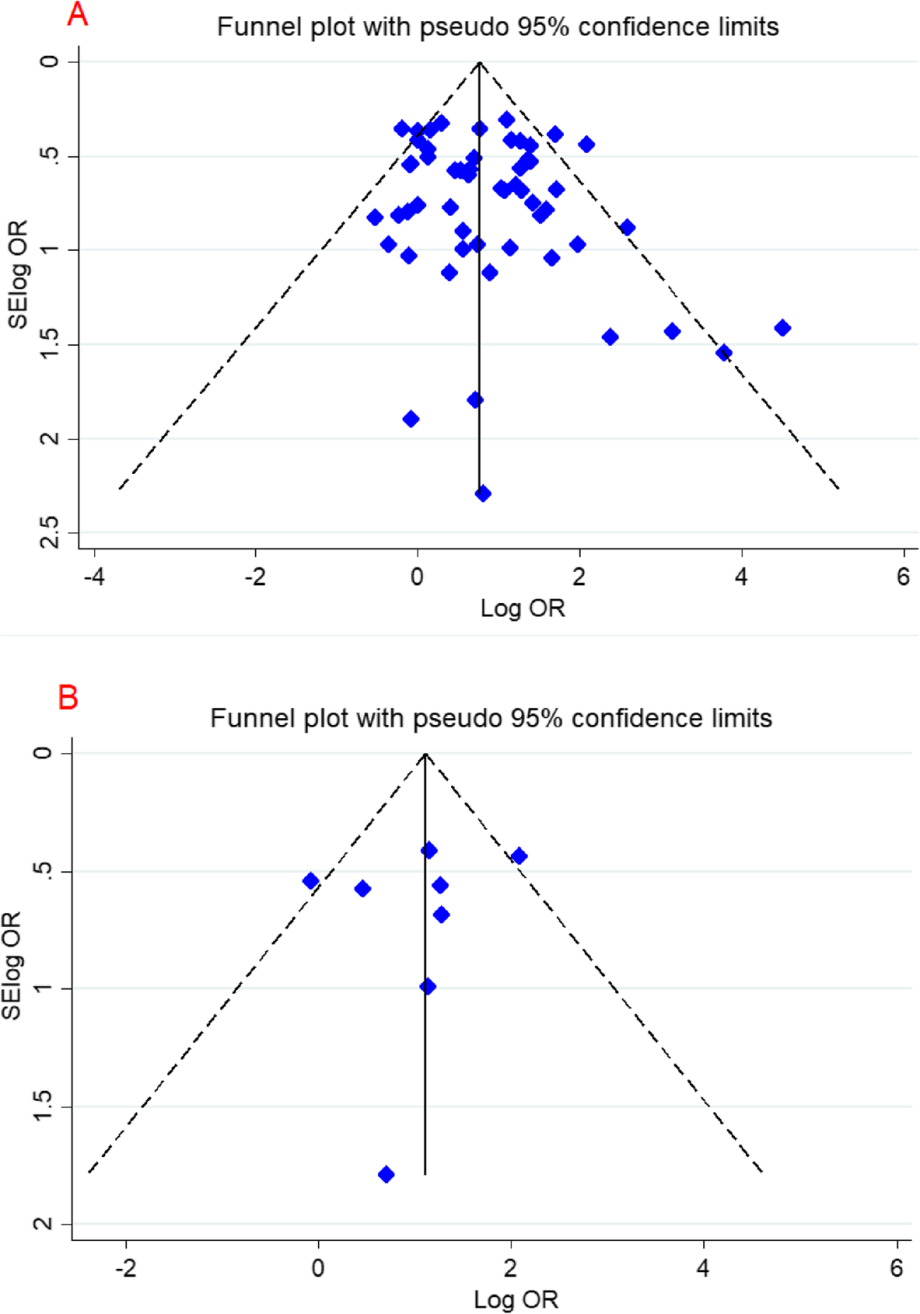
Begg’s funnel plot for publication bias test for the association between FVL 1691G > A mutation and the risk of RPL in the dominant model; **a**:overall population, **b**: Iranian studies . Each point represents a separate study for the indicated association

To check existence of publication bias, Egger’s linear regression and Begg’s funnel plot test were used. The shape of the funnel plots did not disclose obvious asymmetry under all the genotype model of the *FVL* 1691G > A mutation (Fig. 4). Additionally, some degree of heterogeneity was detected in overall population. Therefore, we stratified study by continent and study design to find its potential source.

### Meta-regression analyses

**Fig. 5:**
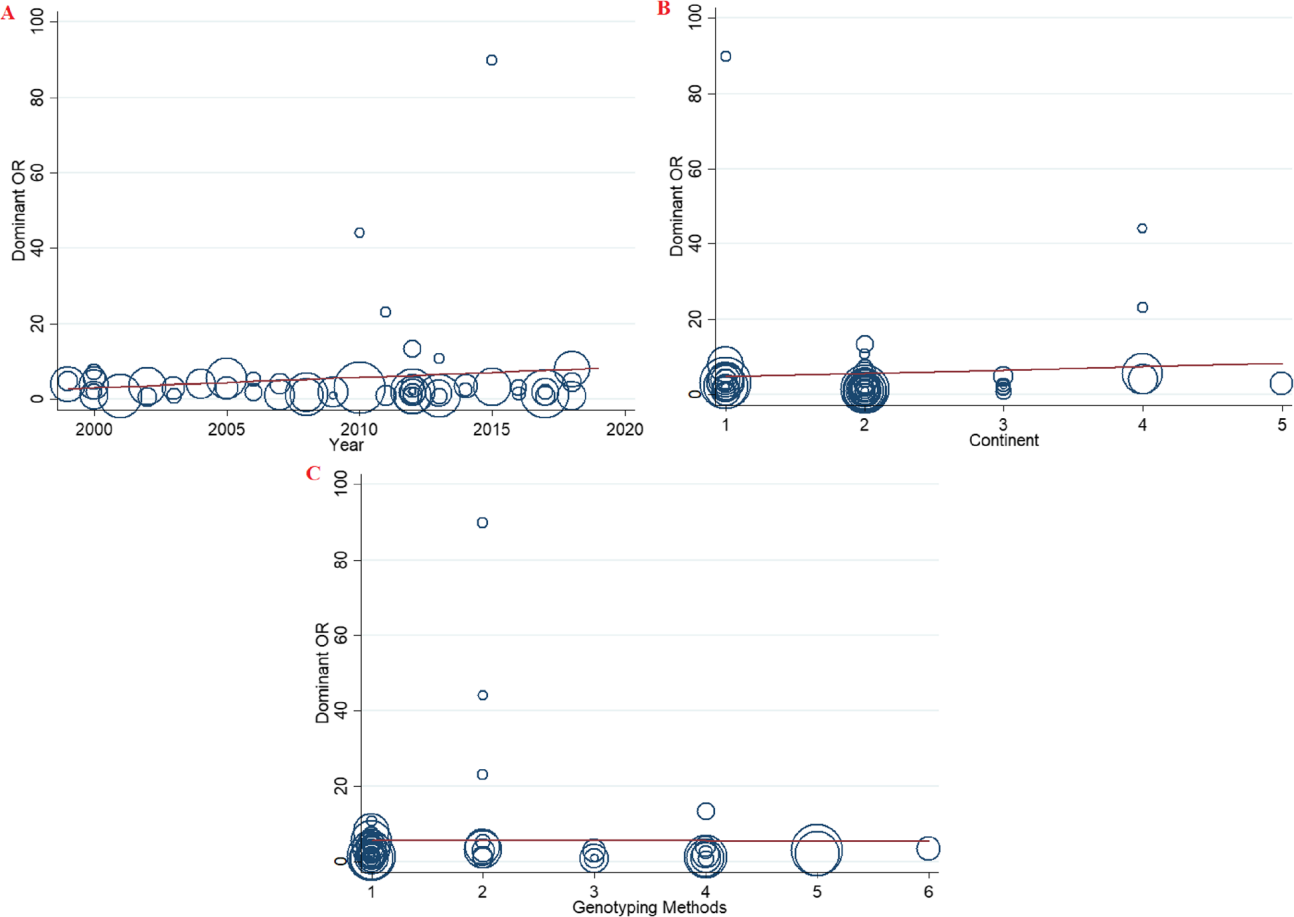
Meta-regression plots of the association between FVL 1691G > A mutation and risk of RPL (Dominant model) based on; **a**: Publication year, **b**: Continent, **c**: Genotyping methods

Meta-regression analyses were performed to explore potential sources of heterogeneity among included studies (Table 4). The findings indicated that none of the expected heterogeneity parameter were the source of heterogeneity (Fig. 5).

**Table 4:** Meta-regression analyses of potential source of heterogeneity

Heterogeneity Factor		Coefficient	SE	T-test	P-value	95% CI	
						UL	LL
**Publication Year**	Dominant	0.296	0.31	0.85	0.39	−0.365	0.905
	Over-Dominant	0.211	0.26	0.79	0.43	−0.325	0.747
	Allelic model	0.159	0.20	0.77	0.44	−0.257	0.576
	GA vs. GG	0.253	0.29	0.86	0.39	−0.341	0.848
**Continent**	Dominant	0.879	1.92	0.46	0.65	−2.99	4.74
	Over-Dominant	0.498	1.63	0.30	0.76	−2.79	3.78
	Allelic model	0.650	1.27	0.51	0.61	−1.90	3.20
	GA vs. GG	0.72	1.80	0.40	0.69	−2.90	4.35
**Genotyping Methods**	Dominant	−0.04	1.35	−0.04	0.97	−2.76	2.66
	Over-Dominant	0.028	1.15	0.02	0.98	−2.29	2.35
	Allelic model	− 0.115	0.89	−0.13	0.89	−1.92	1.68
	GA vs. GG	0.016	1.26	0.01	0.98	−2.52	2.55

### Sensitivity analysis

**Fig. 6:**
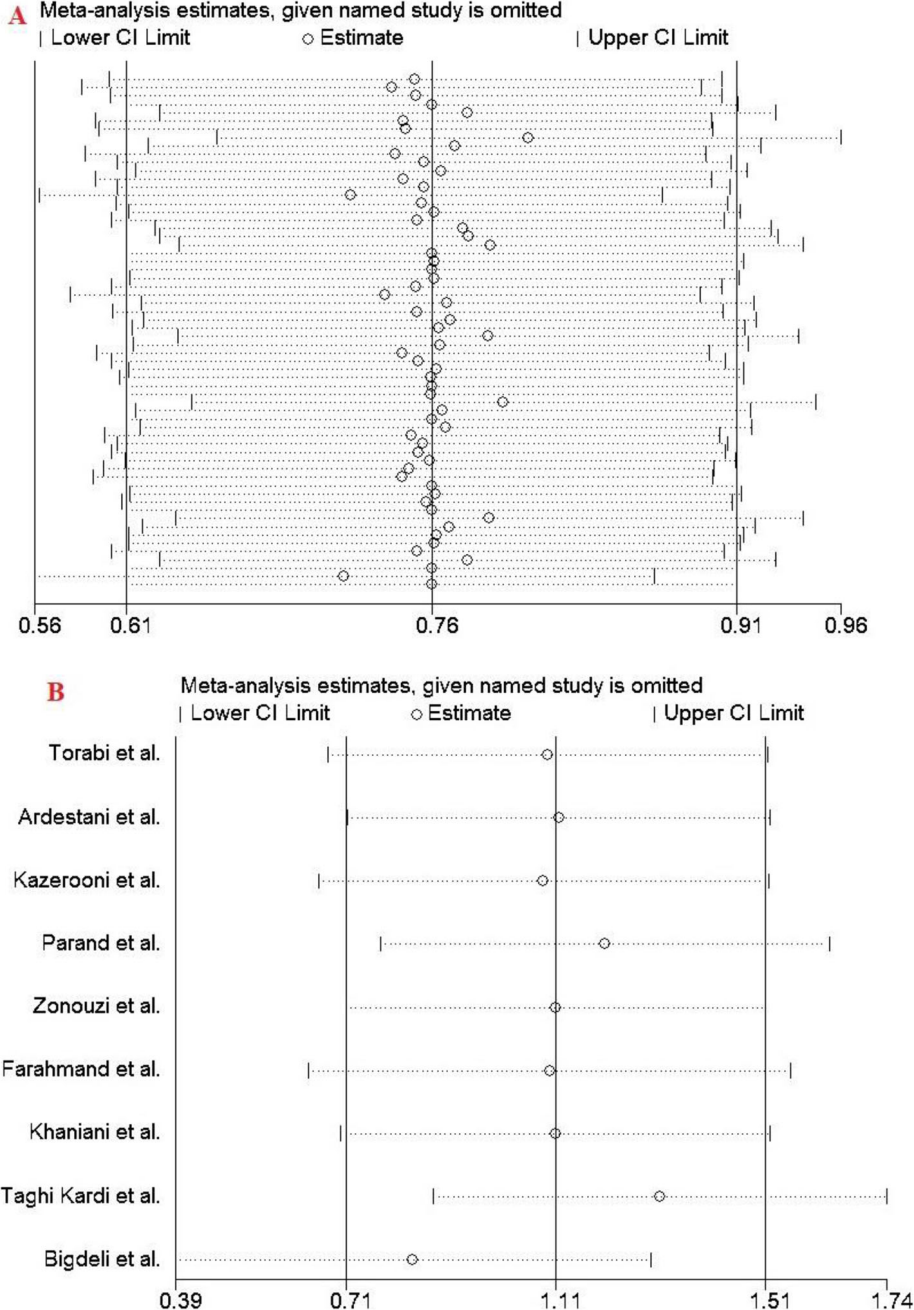
Sensitivity analysis in the present meta-analysis investigates the association of FVL 1691G > A mutation an risk of RPL; **a**: overall population, **b**: Iranian studies

The impact of individual study on pooled OR was evaluated by sequential omission of each studies. The analysis results showed that no individual study significantly affected the pooled ORs under any genotype models of the FVL 1691G > A mutation (Fig. 6).

## Discussion

RPL has been one of the most prevalent obstetric complications, that affect more than 30% of gestations. A remarkable amount of pregnancy losses has been attributed to genetic variations, of which over 50% have been related to chromosomal abnormalities. Several investigations have reported the association of FVL 1691G > A mutation with RPL; that notwithstanding, there have been conflicting results among various ethnicities. The inconsistent results have been attributed to variety in the race of included subjects, different diagnostic criteria of patients, little statistical power, small sample sizes, and the linkage disequilibrium (LD) between various genes and variations [88]. However, meta-analysis strategy provides a pertinent tool to settle the problem of confliction by resolving the limitations of single replication studies, such as limited statistical power and little sample size. Thus, here we conducted the first meta-analysis to find a valid estimation of the association between FVL 1691G > A mutation and risk of RPL.

The FVL 1691G > A mutation is a G-to-A point mutation at nucleotide 1691 in the factor V gene, that results in the single amino-acid replacement Arg506Gln, leading to resistance to be cleaved and, therefore, inactivation by APC and promoted susceptibility to clotting [89, 90]. This mutation enhances the risk of venous thrombosis up to 50–100 times in homozygote carriers [22].

In this meta-analysis, 62 studies, containing 10,410 cases and 9406 controls, were included in quantitative analysis. The analysis of overall population indicated that all genetic comparisons of the FVL 1691G > A mutation, including dominant model (OR = 2.15), over-dominant model (OR = 1.88), allelic model (OR = 2.05), and heterozygote model (OR = 1.97) significantly increased the risk of RPL susceptibility. In 2015, Sergi et al. [91] by including nine studies, containing a total of 2147 women for the FVL mutation, 1305 women with early RPL, and 842 women with no gestational complications, indicated higher carrier frequency of FVL mutation in women with early RPL (OR = 1.68). Moreover, Marcelo and colleagues [92] in 2019 revealed that there was no association between recurrent miscarriage and inherited thrombophilias in patients with polycystic ovarian syndrome, with respect to FVL (OR = 0.74; 95% CI = 0.38 – 1.45; *P* = 0.38), among others. On the other hand, a comprehensive systematic review and meta-analysis in 2016 [93], by exerting 369 articles evaluating 124 polymorphisms of 73 genes, to explore the potential genetic biomarkers for recurrent miscarriage identified increased risk of the disease in the recessive and over-dominant models, but a decreased risk in the dominant and allelic models for FVL 1691G > A mutation, both in overall analysis and subgroup analysis in Caucasians. Our analysis is unique of its type, as it included only patients having RPL diagnosis. Moreover, our subgroup analysis based on the continent of the study population divulged a strong association between FVL 1691G > A mutation and the risk of RPL in Asian, European, and Africa populations, but not in South Americans. It should be noted that among the 62 case-control studies included, 25 studies were in Asia, 26 studies in Europe, 6 studies in South America, 4 studies in Africa, and 1 study in Oceania. Although the subgroup analysis of 6 studies in South America indicated an OR < 1 (which was not significant across all genetic models), all other populations (which made large portion of the studies included) had OR > 1, imply that the South America data had little effect on the pooled effect estimation. The other parameter for subgroup analysis was study design. In this regard, a significant positive association between FVL 1691G > A mutation and the risk of RPL was observed in case-control studies, while cohort studies revealed no such association. The result of this subgroup should interpret with caution because of imbalance between included studies in each group (60 vs. 2).

On the other side, the analysis was also performed in the Iranian population, containing 9 publications with 1409 cases and 1160 controls. The previous meta-analysis in Iranian population by Kamali et al. [94] in 2018, by employing 7 studies, indicated significant increased risk of RPL only in the allelic (OR = 2.252) and dominant models (OR = 2.217). However, our analysis indicated that the measured genetic models, including dominant model (OR = 2.97), over-dominant model (OR = 2.58), and heterozygote model (OR = 2.67, 95%) increased the risk of RPL. The difference between our analysis and the previous one was that we included two more study with higher sample size.

There was a degree of heterogeneity during the overall analysis. From statistical perspective, this heterogeneity describes the variability between included studies and may originate from clinical or methodological heterogeneity, from other unreported, unknown study characteristics, or may be due to chance. Therefore, for finding any sources of heterogeneity and attenuating their effects, we conducted subgroup analysis and weighted meta-regression. Collectively, the results of meta-regression showed that none of the parameters, including publication year, the continent of the study population, and genotyping methods were the expected source of heterogeneity. However, subgroup analysis reduced heterogeneity in all groups and explained part of the observed heterogeneity expect Asians and studies with cohort design. Furthermore, the other way of dealing with statistical heterogeneity, which we used in our analysis, was to incorporate “Random” term to account for it in a random-effects. Random effect model typically produces more conservative estimates of the significance of a result (a wider confidence interval). As it gives proportionately higher weights to smaller studies and lower weights to larger studies than fixed effect analysis.

To address the limitations in the current meta-analysis, it should be stated that, first our literature search was limited to only studies published in English language. Second, there was a degree of heterogeneity during the overall analysis. But not in all subgroup analyses, indicating the role of genetic diversity and other confounders in susceptibility to RPL. Third, as this meta-analysis a crude estimation of the association between FVL 1691G > A mutation and the risk of RPL, thus the roles of age, paternal genetic impression, environmental factors, and the effect of gene-gene interactions in conferring the susceptibility risk to RPL were neglected.

Considering all the facts, this meta-analysis, the first one of its type to our best knowledge, retrieved 62 studies, encompassing 10,410 cases and 9406 health controls, to find a consistent result of the association between FVL 1691G > A mutation and risk of RPL. Our results indicated statistically significant increased risk of RPL in the overall analysis. The increased susceptibility to RPL was also observed in Iranian, Asian, European, Africa populations, and studies with case-control design, but not in South Americans and studies with cohort design. Further experiments, alongside with inclusion of additional studies with large sample sizes, should consider the role cofounders in susceptibility to RPL.

## Data Availability

All data that support the conclusions of this manuscript are included within the article.

## Abbreviations

FVL: Factor V leiden
RPL: Recurrent pregnancy loss
MTHFR: Methylenetetrahydrofolate reductase
CI: Confidence interval
OR: Odds ratio
SNP: Single-nucleotide polymorphism
PRISMA: Preferred reporting items for systematic reviews and meta-analyses
NOS: Newcastle–Ottawa scale
HWE: Hardy–Weinberg equilibrium
